# Editorial: Unraveling Sleep and Its Disorders Using Novel Analytical Approaches

**DOI:** 10.3389/fnins.2022.924359

**Published:** 2022-05-18

**Authors:** Gonzalo C. Gutiérrez-Tobal, Leila Kheirandish-Gozal, David Gozal, Roberto Hornero

**Affiliations:** ^1^Biomedical Engineering Group, University of Valladolid, Valladolid, Spain; ^2^Centro de Investigacion Biomedica en Red en Bioingenieria, Biomateriales y Nanomedicina (CIBER-BBN), Valladolid, Spain; ^3^Department of Child Health, Child Health Research Institute, The University of Missouri School of Medicine, Columbia, MO, United States

**Keywords:** sleep apnea, cardio-pulmonary coupling, insomnia, sleep deprivation, sleep disruption, depression

Despite significant and meritorious research efforts over the last decades, the functions and evolutionary determinants of sleep remain one of the mysterious and relatively unexplored dimensions of physiology (Krueger et al., 2016). The recipe for deciphering such an attractive challenge in biology and medicine most probably includes ingredients such as unraveling the complexity of brain functioning during sleep (Olbrich et al., 2011), the specific roles of sleep in coordination of other body systems (Penzel et al., 2016), or defining the specific cellular and system-related pathways that regulate or are regulated by sleep. In addition, the already established and novel techniques and technological tools aiming to explore sleep-related systems, e.g., electroencephalogram (EEG), magnetoencephalogram, positron emission tomography, functional magnetic resonance image (fMRI) and several others in the case of the brain, have their own limitations and advantages (Huster et al., 2012).

This Research Topic aimed to be an overarching framework and instigator for those sleep-related studies presenting novel conceptual approaches and disruptive ideas in the field. Manuscripts submitted for this special issue represent high scientific contributions to this topic. The only drawback in the Editors' opinion is that most of the studies focused on sleep disorders rather than on deep and sophisticated analyses of physiological sleep. This Research Topic might however stimulate further research in this direction.

Among the studies finally incorporated to the Research Topic, four of them focused on sleep apnea and cardio-pulmonary coupling, four additional articles focused on insomnia, sleep deprivation, and micro-sleep episodes, one focused on sleep disruption and, finally, another one focused on depression. Consistent with these topics, we have organized the rest of the document in four sections.

## Sleep Apnea and Cardio-Respiratory Coupling During Sleep

The first study we present is an excellent example of the close relationship between sleep research and the emerging technologies and analytical techniques. Kelly et al. used at-home recordings of overnight mandibular movements to validate an automated machine-learning approach based on random forest aimed at diagnosing sleep apnea in adult subjects. Very high accuracy performances were reported for several apnea-hypopnea cutoffs, thus highlighting the approach as a promising alternative to the standard and labor-intensive diagnostic methodologies.

Karhu et al. explored further on the implications of the oxygen desaturation episodes in the context of sleep apnea evolution. They analyzed 805 adults with mild sleep apnea from the well-known Sleep Heart Health Study to show how the characteristics of the episodes, rather than their counts number, may be predictive of sleep apnea worsening 5 years later.

Gutiérrez-Tobal et al. analyzed the overnight electroencephalogram (EEG) from 294 children to find sleep apnea-related effects, including cognitive implications. A correlation network analysis on spectral features from the EEGs, clinical variables, and cognitive scores unraveled both general patterns and specific associations that showed severity-dependent sleep apnea features.

Finally, Al Ashry et al. presented a very interesting review on cardio-pulmonary coupling during sleep. They showed how distinct patterns are observed in the cardio-pulmonary spectrogram, built with heart rate variability and derived respiratory signals, according to different sleep stages, sleep apnea endotypes and phenotypes, insomnia, and other sleep-related conditions.

### Insomnia, Sleep Deprivation, and Micro-Sleep Episodes

Vaziri et al. presented a “Hypothesis and Theory” article regarding insomnia. Particularly, they propose a novel conceptual framework for the cognitive implications of insomnia in a search for more personalized clinical interventions. The authors argue that their methodology could pave the way to conceptual frameworks for other cognitive problems.

Xu et al. combined fMRI data, a network centrality analysis, and machine learning (support vector machines) to predict sleep deprivation vulnerability. They report remarkable results in the task while pointing out the brain regions that contribute the most to such susceptibility in performance.

Another sleep deprivation study is presented by Xiong et al.. The authors evaluate the effect of the arousal enhanced drug Modafinil to mitigate the cognitive decline after sleep deprivation. They used a rat model to report that the drug suppresses neuronal pyroptosis and inflammation associated to sleep deprivation.

Finally, a study on micro-sleep episodes detection is presented by Malafeev et al.. The authors used a combination of deep-learning techniques (convolutional neural networks and recurrent neural networks) to identify episodes of wakefulness, micro-sleep episodes, micro-sleep candidates, and drowsiness on the EEG and electro-oculogram of 76 patients with excessive daytime sleepiness. Micro-sleep episodes and wakefulness were reported to be highly separable, while significant overlap of these with micro-sleep candidates and drowsiness were also shown.

### Sleep Disruption

A very interesting review on new approaches to improve sleep disruption definitions is presented by Lechat et al.. They start by pointing out the limits of current standards and then go over some of the latest discoveries and techniques that have helped gain insights into sleep disruption. The authors highlighted alterations in EEG (in slow waves, K-complexes, or sleep spindles), oxygen saturation (hypoxic burden), and other respiratory signals (low arousal threshold, high loop gain, etc.), while acknowledging automatic processing techniques such as spectrogram, signal coupling measures, and environmental factors.

### Depression

A study on the brain activity alterations during sleep caused by depression is presented by Lian et al.. The authors analyzed the sleep EEG of 25 healthy controls and 26 depressed patients using symbolic phase transfer entropy as a measure of effective connectivity between electrodes, within the common spectral bands, and in the different sleep stages. Results reported show how the main way of information transition during sleep is attenuated in depressed patients.

Altogether, these 10 studies underline the impact of sleep in our lives, the need for increasing the research efforts on this topic, as well as on how to develop and apply new powerful tools to do it.

## Author Contributions

GG-T, LK-G, DG, and RH reviewed the submissions, wrote the editorial, and approved it for publication. All authors contributed to the article and approved the submitted version.

## Funding

This work was supported by the Ministerio de Ciencia, Innovación y Universidades and the European Regional Development Fund (FEDER) under projects PID2020-115468RB-I00, and PDC2021-120775, by the European Commission and FEDER under project Análisis y correlación entre el genoma completo y la actividad cerebral para la ayuda en el diagnóstico de la enfermedad de Alzheimer (Cooperation Programme Interreg V-A SpainPortugal POCTEP 20142020), and by CIBER-BBN (ISCIII), cofunded with FEDER funds. DG was supported by the United States National Institutes of Health AG061824, and by Tier 2 and TRIUMPH grants from the University of Missouri.

## Conflict of Interest

The authors declare that the research was conducted in the absence of any commercial or financial relationships that could be construed as a potential conflict of interest.

## Publisher's Note

All claims expressed in this article are solely those of the authors and do not necessarily represent those of their affiliated organizations, or those of the publisher, the editors and the reviewers. Any product that may be evaluated in this article, or claim that may be made by its manufacturer, is not guaranteed or endorsed by the publisher.
